# Solonamide B Inhibits Quorum Sensing and Reduces *Staphylococcus aureus* Mediated Killing of Human Neutrophils

**DOI:** 10.1371/journal.pone.0084992

**Published:** 2014-01-08

**Authors:** Anita Nielsen, Maria Månsson, Martin S. Bojer, Lone Gram, Thomas O. Larsen, Richard P. Novick, Dorte Frees, Hanne Frøkiær, Hanne Ingmer

**Affiliations:** 1 Department of Veterinary Disease Biology, Faculty of Health and Medical Sciences, University of Copenhagen, Copenhagen, Denmark; 2 Center for Microbial Biotechnology, Department of Systems Biology, Technical University of Denmark, Lyngby, Denmark; 3 Skirball Institute of Biomolecular Medicine, New York University School of Medicine, New York, New York, United States of America; The Scripps Research Institute and Sorrento Therapeutics, Inc., United States of America

## Abstract

Methicillin-resistant *Staphylococcus aureus* (MRSA) continues to be a serious human pathogen, and particularly the spread of community associated (CA)-MRSA strains such as USA300 is a concern, as these strains can cause severe infections in otherwise healthy adults. Recently, we reported that a cyclodepsipeptide termed Solonamide B isolated from the marine bacterium, *Photobacterium halotolerans* strongly reduces expression of RNAIII, the effector molecule of the *agr* quorum sensing system. Here we show that Solonamide B interferes with the binding of *S. aureus* autoinducing peptides (AIPs) to sensor histidine kinase, AgrC, of the *agr* two-component system. The hypervirulence of USA300 has been linked to increased expression of central virulence factors like α-hemolysin and the phenol soluble modulins (PSMs). Importantly, in strain USA300 Solonamide B dramatically reduced the activity of α-hemolysin and the transcription of *psma* encoding PSMs with an 80% reduction in toxicity of supernatants towards human neutrophils and rabbit erythrocytes. To our knowledge this is the first report of a compound produced naturally by a Gram-negative marine bacterium that interferes with *agr* and affects both RNAIII and AgrA controlled virulence gene expression in *S. aureus*.

## Introduction

*Staphylococcus aureus* is a serious human pathogen that causes a variety of diseases, such as skin and soft tissue infections, bacteremia, and toxic shock syndrome [1], [2]. The organism is well known for its ability to develop resistance to a wide range of antibiotics and in consequence only few treatment options are now available for the most resistant strains [3]. Resistance to methicillin is particularly widespread, and nosocomial infections with methicillin resistant *S. aureus* (MRSA) strains are one of the most serious risk factors associated with hospitalization [4]. While the hospital associated *S. aureus* strains are generally opportunistic pathogens incapable of infecting healthy individuals [5] a more aggressive group of strains have emerged since the early 1990s that is both highly virulent and transmissible giving rise to infections in the community, thus termed community associated, methicillin resistant strains (CA-MRSA). The CA-MRSA strains belong to several sequence types with USA300 (ST8) being the most common in the US [6], [7]. Importantly, these strains are able to infect healthy individuals often giving rise to skin and soft tissue infections that in some instances may turn out to be lethal [8], [9].

Two of the most important virulence factors of CA-MRSA are α-hemolysin [10] and the phenol soluble modulins, the PSMs [11]. α-hemolysin is a pore forming α-toxin that lyses immune cells such as phagocytes, erythrocytes, and lymphocytes [12]. Also, α-hemolysin is required for *Staphylococcus aureus* phagosomal escape after internalization in a cystic fibrosis epithelial cell line [13]. PSMs are a class of secreted surfactant-like, amphipathic, alpha-helical staphylococcal peptides and they are remarkable at recruiting, activating and subsequently lysing human neutrophils. There are four alpha-types and two beta-type PSMs. The alpha-type PSMs are about 20–25 amino acids in length, and especially PSMα3 is responsible for the lysis of human neutrophils. The beta-type PSMs are longer, about 40–45 amino acids and lack cytolytic activity [11], [14]. Neutrophils constitute an essential part of the innate immune system, as they hold strong phagocytotic activity and are recruited to the site of infection in high numbers [15]. Thus, the production of PSMs is critical for the ability of *S. aureus* to evade the host immune system and as such is determining for the outcome of the infection [11].

The exceptionally high expression of toxins and exoenzymes by CA-MRSA strains such as USA300 relies on the *agr* two-component quorum sensing system encoded by *agrACDB* [16], [17]. With increasing cell density, autoinducing peptides (AIPs) produced by *agrB* and *agrD* [18] accumulates in the extracellular space and binds to the sensor histidine kinase, AgrC. Upon AIP binding, AgrC activates the response regulator, AgrA that stimulates transcription of a small regulatory RNA, RNAIII, responsible for the expression of α-hemolysin and other toxins in the late exponential growth phase [19], [20]. AIPs consist of seven to nine amino acids in which the central cysteine residue is covalently linked to the terminal carboxylate, forming a thiolactone ring [21], [22]. Between strains, AIP structure varies giving rise to at least four AIP classes where an AIP activates *agr* of strains only belonging to the same class but represses *agr* of the other classes [20]. In contrast to most toxins, expression of the PSMs is controlled directly by AgrA that binds to the promoter region of the *psmα* and *psmβ* operons respectively and activates transcription [23].

In common to both community and hospital associated *S. aureus* infections, resistance to antibiotics is an increasing problem and we urgently need new approaches to prevent and treat *S. aureus* infections caused by resistant strains [24]–[27]. Anti-virulence compounds may offer an alternative to antibiotics, as they target the expression or activity of virulence factors, rather than growth or viability [28], [29]. Examples of anti-virulence therapies include neutralization of toxins using antibodies [30], prevention of adhesion [31] or interference with virulence gene regulation [32]. Advantages to such approaches may be that the host microbiota is left unharmed and that there is likely to be less selection of drug-resistance [33]. In a search for compounds that reduce virulence gene expression in *S. aureus* we discovered that Solonamide B which is produced by a marine *Photobacterium halotolerans*, reduces expression of α-hemolysin and *rnaIII* and increases expression of *spa* encoding protein A in both strain 8325-4 and USA300 [34]. The purpose of the present study was to determine the mode of action and the extent to which RNAIII and AgrA controlled virulence factors were affected. We show here that Solonamide B is likely to interact directly with the *agr* quorum sensing system and that the activity of the compound is influencing expression of both RNAIII and AgrA controlled toxins.

## Materials and Methods

### Bacterial Strains and Growth Conditions

*S. aureus* strains used in this study included: Strain NCTC 8325 (RN1), and 8325-4 [35] and FPR3757 USA300 a multidrug resistant CA-MRSA isolate implicated in outbreaks of skin and soft tissue infection [36]. For competition assays PC203 (*S. aureus* 8325-4, *spa::lacZ*) [38] and RN6911 (*S. aureus* 8325-4 Δ*agr*) [19] were also employed. During the study we constructed a series of *lux*-reporter strains. Plasmid from *S. aureus* strain RN9723 containing the P3*::luxABCDE* construct (R. Novick) was phage transduced into *S. aureus* strains with different *agr* group backgrounds (RN1 8325 *agr* gr.I, RN6607 *agr* gr.II, MOZ53 *agr* gr.III (clinical isolate), RN4850 *agr* gr.IV, R. Novick) obtaining strain AN1: (RN1 8325 *agr* gr.I, P3::*luxABCDE*, CM^R^), AN2: (RN6607 *agr* gr.II, P3*::luxABCDE*, CM^R^), AN3: (MOZ53 *agr* gr.III,P3*::luxABCDE*, CM^R^) and AN4: (RN4850 *agr* gr.IV, P3*::luxABCDE*, CM^R^. Strain RN10829/pEG11 [40], a β-lactamase reporter strain substituting the native *agr* locus with a chromosomal integration of P2-*agrA* and P3-*blaZ* and a plasmid from which a constitutive active variant of AgrC (*agrC-I-R238H*) is expressed, was used to assess AgrC-dependent effects of Solonamide B. Strain RN6911 [19] was used to generate bacterial supernatant not containing auto-inducing peptide (AIP) (strain 8325-4 was used to obtain supernatant containing AIP gr.I.). Unless otherwise stated, bacteria were grown in Tryptone Soya Broth (TSB), Oxoid, (1∶10 volume/flask ratio), at 37°C with shaking at 200 rpm.

### Activity of Solonamide B in WT and Constitutive Active AgrC reporter Strains

Plasmid p*agrC-I-WT* was generated by site-directed mutagenesis (QuikChange Site-Directed Mutagenesis Kit, Agilent Technologies) using primers 5′-aacaacgaaatgcgcaagttccgtcatgattatgtcaatatcttaa-3′ (AgrCHtoR_Fwd) and 5′-ttaagatattgacataatcatgacggaacttgcgcatttcgttgtt-3′ (AgrCHtoR_Rev) containing the desired nucleotide substitution (underlined) and plasmid pEG11 [40] as template following the manufacturer’s instructions. Correct reversion to AgrC-I-WT was confirmed by sequencing and by displaying wild-type characteristics, i.e., an AIP-I-dependent induction of β-lactamase activity in strain RN10829. Cultures of reporter strains, containing either p*agrC-I-WT* or pEG11, were diluted to OD_600_ 0.05 in fresh TSB (without antibiotics) and grown to OD_600_ 0.4–0.5 (37°C, 225 rpm) followed by addition of 5 or 10 µg/mL Solonamide B (final concentration) or DMSO (solvent) and 1/10 the volume of spent medium containing or free from AIP-I. Bacterial samples were taken at indicated time points, cooled instantly in an ice-water bath and frozen at −80°C. The respective culture OD_600_ was recorded. β-lactamase activity of samples was subsequently determined by the nitrocefin hydrolysis method [21]. Briefly, 50 µL cell culture was added an equal volume of 0.2 mM nitrocefin (Oxoid) in 0.1 M phosphate buffer (pH 7.0) and measuring changes in OD_486_ over time in a PowerWave XS microplate reader (BioTek) at 37°C. Activities were calculated as arbitrary units based on respective conversion velocities (V_max,_ ΔOD_486_/time) normalized to the sample cell densities.

### Competition Assay

The reporter assay were made according to Nielsen et al. (2010) [37] using the *S. aureus* 8325-4 derived *spa::lacZ* reporter strain [38] or strain RN10829 carrying p*agrC-I-WT*. Sterile filtered supernatants from ON-cultures were made from *S. aureus* 8325-4 (wt) [35] and *S. aureus* 8325-4 Δ*agr* [19] grown in TSB. For AIP competition experiments using the P3-*blaZ* reporter, 5 µg/mL Solonamide B was challenged by addition of a fixed 1/5 volume of culture supernatant, adjusted to contain 5%, 13%, or 20% AIP-I-containing supernatant (final concentration), and the effect was monitored in the AgrC-I-WT reporter strain. Solonamide B were purified from cultures of the marine bacterium *Photobacterium halotolerans* as described by Mannson et al. (2011) [34].

### Effect of Solonamide B on *agr*-group I, II, III and IV

Strain AN1, AN2, AN3 and AN4 were used for testing the effect of Solonamide B on *S. aureus* with different *agr* backgrounds (*agr* gr. I, II, III and IV). Cells were grown until OD_600_ = 0.4 and diluted 10 fold before distribution into a 96 well microtiter plate (100 µL per well). Solonamide B dissolved in DMSO was added to the microtiter well to a final concentration of 5 and 10 µg/ml. The plate was incubated at 37°C in a microplate reader with shaking every 10 minutes. Luminescence was recorded after 2½ hour incubation at OD_600_ = 0.3 in microtiter plates. For *agr* group III luminescence was recorded every 20 minutes in the absence of shaking.

### Rabbit Erythrocyte Hemolysis Assay

1% suspension of rabbit erythrocytes were prepared by spinning 2 ml of rabbit blood at 3000 *g* for 10 min at 10°C, where after supernatant was removed. Pellet was dissolved in 2 mL of PBS containing 0.1% BSA (bovine serum albumin). Centrifugation and washing was repeated until the supernatant was clear (approx 3 times). To prepare a 1% erythrocyte solution 100 µL erythrocytes were dissolved in 10 ml PBS containing 0.1% BSA. To prepare culture supernatants *S. aureus* USA300 (FPR3757) cultures were spun at 5000 *g* for 10 minutes to pellet the bacteria. The supernatant was transferred to new eppendorf tubes, and dilution rows (2-fold dilutions) were made in TSB. 100 µL of 1% erythrocytes suspension was added to 100 µl culture supernatant in a 96 well microtiter plate and incubated for 30 minutes at 37°C and 30 minutes at 4°C. 0.2% Triton X in TSB (final concentration of 0.1%) was used as a positive control, and TSB was used as a negative control. The microtiter plate was spun at 2000 rpm for 2 minutes, supernatant was transferred carefully to a new plate and read at OD_450_.

### Transcriptional Analysis of *agr*A and *psm*α by Northern Blot

Northern blot analysis was performed as described previously [39]. The strains used were *S. aureus* 8325-4 and FPR 3757 USA300, and were grown in Erlenmeyer flasks, shaking at 185 rpm. Growth was monitored by measuring optical density at OD_600_. Start inoculum was OD_600_ = 0.03. Solonamide B was added at OD_600_ = 0.4. Samples for RNA purification were taken at OD_600_ = 0.7 and 1.7. Probes targeting AgrA and PSMα transcripts were amplified by PCR using the primers AgrA fwd: 5′ ctgataatccttatgaggtgc 3′, AgrA rev: 5′ cgatgcatagcagtgttc 3′, PSMα rev: 5′ tatcaaaagcttaatcgaacaattc 3′ and PSMα fwd:5′ ccccttcaaataagatgttcatatc 3′ (from TAG Copenhagen A/S, Denmark) resulting in probe lengths of 584 bp (AgrA) and 176 bp (PSMα) respectively.

### *S. aureus* lysis of Human Neutrophils

Sterile filtered supernatants from 7 h and 22,5 h *S. aureus* LAC/FPR3757 (USA300) cultures grown in 10 mL TSB media/100 mL Erlenmeyer flasks, with a start inoculum OD_600_ = 0.02 and either DMSO (control) or Solonamide B (5 and 10 µg/mL) added to the cultures were tested for their capacity to lyse human neutrophils. Bacterial supernatants were collected from three independent experiments on different days, and stored at −20°C until used. Lysis of human neutrophils was determined using Cytotoxicity Detection Kit^plus^ (LDH) from Roche (measuring lactate dehydrogenase release), manufacturer’s protocol was followed. Supernatants diluted 3- and 9-fold and undiluted supernatants were tested. Neutrophil lysis was determined spectrophotometrically at A490 nm-A630 nm after 1 h incubation at 37°C of 100 µL neutrophils (50,000 cells per well)+50 µL supernatant/TSB in microtiter wells. Data is a representative of 3 biological replicates, and each supernatant was tested at least in replicate wells. Neutrophils were isolated by Ficoll-hypaque purification from fresh heparinized human blood from healthy volunteers according to the regulations of National Committee on Health Research Ethics in Denmark. Using blood for assessment of effects of microorganisms or microbial components does not require approval from the committee (law no 593 of June 14, 2011). Blood was mixed with equal volume of dextran (3%)/saline (0.9%), and incubated at room temp. for 20 min. The upper leukocyte rich layer was saved and centrifuged 10 min at 5°C at 250 *g*. Pellet was resuspended in 0.9% NaCl (equal to start volume). Ficoll-hypaque was gently layered under the cell suspension and centrifuged for 40 min. The red blood cells present in the pellet were lysed and cleared by washing with ice cold 0.2% NaCl for 30 sec. followed by adding 1.6% NaCl and centrifuging for 6 min at 250 *g* until colorless pellet was obtained and neutrophils were resuspended in ice-cold PBS/glucose (10 mM).

## Results

### Solonamide B Interferes with *agr* Activation

**Figure 1:**
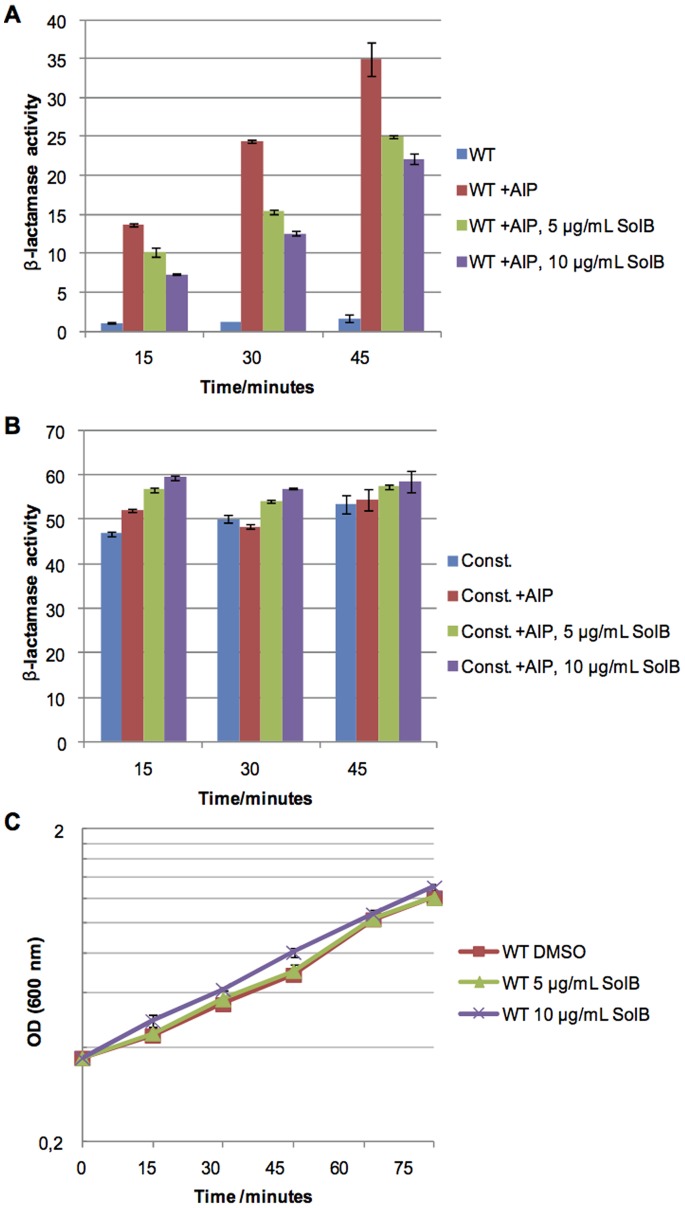
Solonamide B does not influence RNAIII expression in the presence of a constitutively active AgrC. Cultures of RN10829 (P2-*agrA*; P3-*blaZ*) containing either p*agrC-I-WT* (A, denoted WT) or pEG11 (B, denoted Const.) were grown to OD_600_ 0.4–0.5 where 1/10 a volume of AIP-containing supernatant and 5 µg/mL or 10 µg/mL Solonamide B (SolB) or DMSO was added. Controls not containing AIP were included to confirm the inducible and constitutive nature of reporters in A and B, respectively. Samples were obtained at various time points after addition and were analyzed for β-lactamase activity (P3 expression). A single representative experiment is depicted with bars representing the mean +/− standard error of the mean (SEM) from triplicate determinations of β-lactamase activity. Growth monitored for the WT strain confirms that the reduction in P3 expression is not due to growth impairment (C).

From the marine bacterium *Photobacterium halotolerans* we recently identified a cyclodepsipeptide, Solonamide B that interferes with virulence gene expression in *S. aureus* without inhibiting growth. The reduction in *hla* and RNAIII expression and increase in *spa* transcription provided circumstantial evidence that Solonamide B may interfere with *agr* activation [34]. To address this issue we examined the effect of the compound on RNAIII expression in a strain carrying an AgrC variant with a substitution at R238H in the histidine kinase domain that allows AgrC to be active independently of AIPs [40]. If, indeed, solonamide B acts through AgrC we predicted that RNAIII expression in cells carrying the constitutive variant would be unaffected by the compound in comparison to cells expressing the wild type AgrC. At solonamide B concentrations not affecting growth RNAIII (fig. 1C) promoter (P3) activity was monitored in strain RN10829 carrying a chromosomal P3*::blaZ* reporter fusion in addition to plasmids p*agrC-I-WT* or pEG11 expressing wild type or constitutive AgrC, respectively. Since this strain does not produce AIP, *agr* was induced by addition of 1/10 a volume of spent medium obtained from wild type 8325-4 cells in stationary growth phase. We found that in the presence of the constitutive AgrC (fig. 1B), Solonamide B did not affect the P3 promoter activity at concentrations that reduced RNAIII expression significatly in cells expressing wild type AgrC (fig. 1 A). Thus, our results show that the effect of Solonamide B on virulence gene expression is mediated via interactions with AgrC. Furthermore, we noted a tendency towards a dose-dependency on Solonamide B (fig. 1A) suggesting competitive activity.

**Figure 2:**
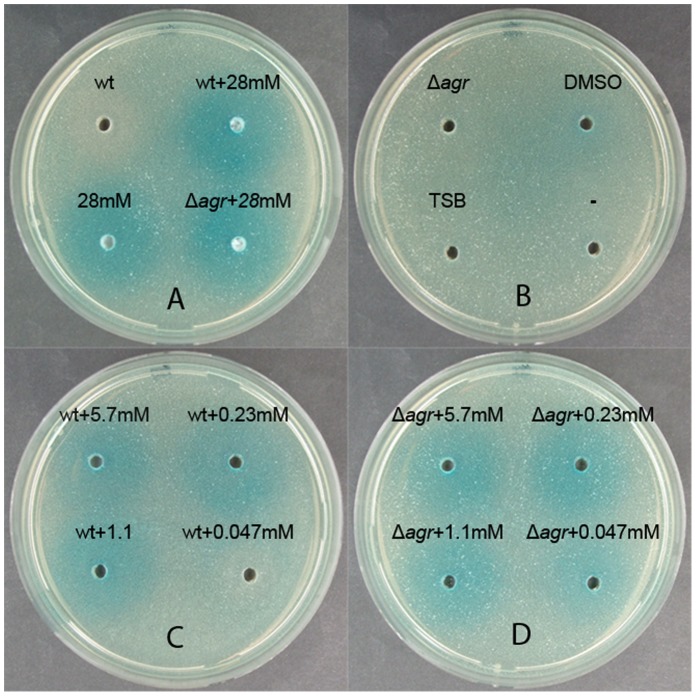
Solonamide B competitively interferes with AIP binding to AgrC. Agar plates containing the *spa-lacZ* reporter strain PC203 were supplied with 10 µl DMSO containing 0, 0.8, 4, 20, 100 and 500 µg Solonamide in DMSO and 20 µl of supernatant of overnight culture of 8325-4 (indicated as “WT”) or RN6911, Δ*agr* mutant cells (indicated as “Δ*agr”*) or TSB medium resulting in no (B) or the indicated concentrations of solonamide B (A, C, D).

**Figure 3:**
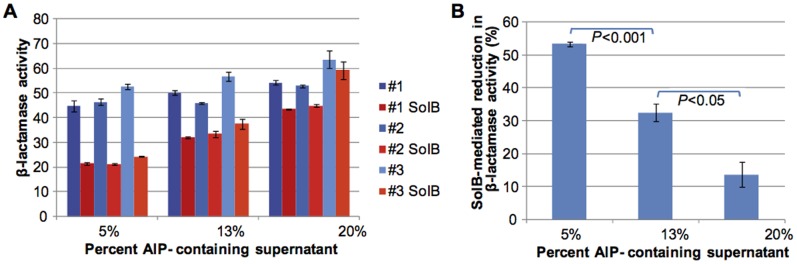
Assessment of competitive action of Solonamide B in P3-*blaZ* reporter strain. The inhibitory activity of 5 µg/mL Solonamide B (SolB) on RNAIII expression controlled by wild type AgrC was assessed when challenged by increasing concentrations of AIP. β-lactamase activity was determined with and without Solonamide B for three independent cultures (#1–#3) 30 minutes after addition of Solonamide B and induction by AIP. Bars represent means +/− SEM from triplicate activity determinations (A). Average inhibition obtained by Solonamide B at increasing AIP concentrations. Bars represent the mean (+/− SEM) reduction in β-lactamase activity observed from the three biological replicates (B). *P*-values between AIP treatments were calculated by Student’s t-test (two-sided).

To address if Solonamide B interferes competitively with AIP binding to the AgrC receptor we added varying amounts of Solonamide B to wells in our reporter assay also containing fixed amounts of culture supernatant from either stationary phase 8325-4 cells or from *agr* mutant cells (fig. 2). Here, we observed that a fixed concentration of solonamide B induced *spa* expression of the incorporated reporter strains both in the presence of spent medium of wild type and *agr* mutant cells thus indicating inhibition of *agr* activity (fig. 2A). When reducing the amount of solonamide B only the lowest concentration (corresponding to 0.05 mM in the well) was unable to induce *spa* expression in competition with spent medium of wild type cells (fig. 2C). However, at the same solonamide B concentration we did see expression of *spa* when competed with supernatant obtained from *agr* mutant cells lacking AIP production thus confirming that solonamide B competes with AIP for AgrC binding (fig. 2D). A more quantitative measure of the competition was obtained with the P3-*blaZ* reporter strain exposed to a fixed concentration of Solonamide B (5 µg/mL) and dilutions of the spent supernatant from stationary phase, wild type cells. The results (fig. 3) show that 50% inhibition was reached when challenged by 5% supernatant, which is comparable to the reduction in P3 activity observed for 10 µg/mL Solonamide B in 10% supernatant (fig. 1A). By increasing the AIP activator concentration a gradual and significant reduction in Solonamide B inhibition is evident, clearly showing the AIP-competitive AgrC-inhibitory activity of Solonamide B (fig. 3).

### Solonamide B Inhibits more than One *agr* Specificity Group

**Figure 4:**
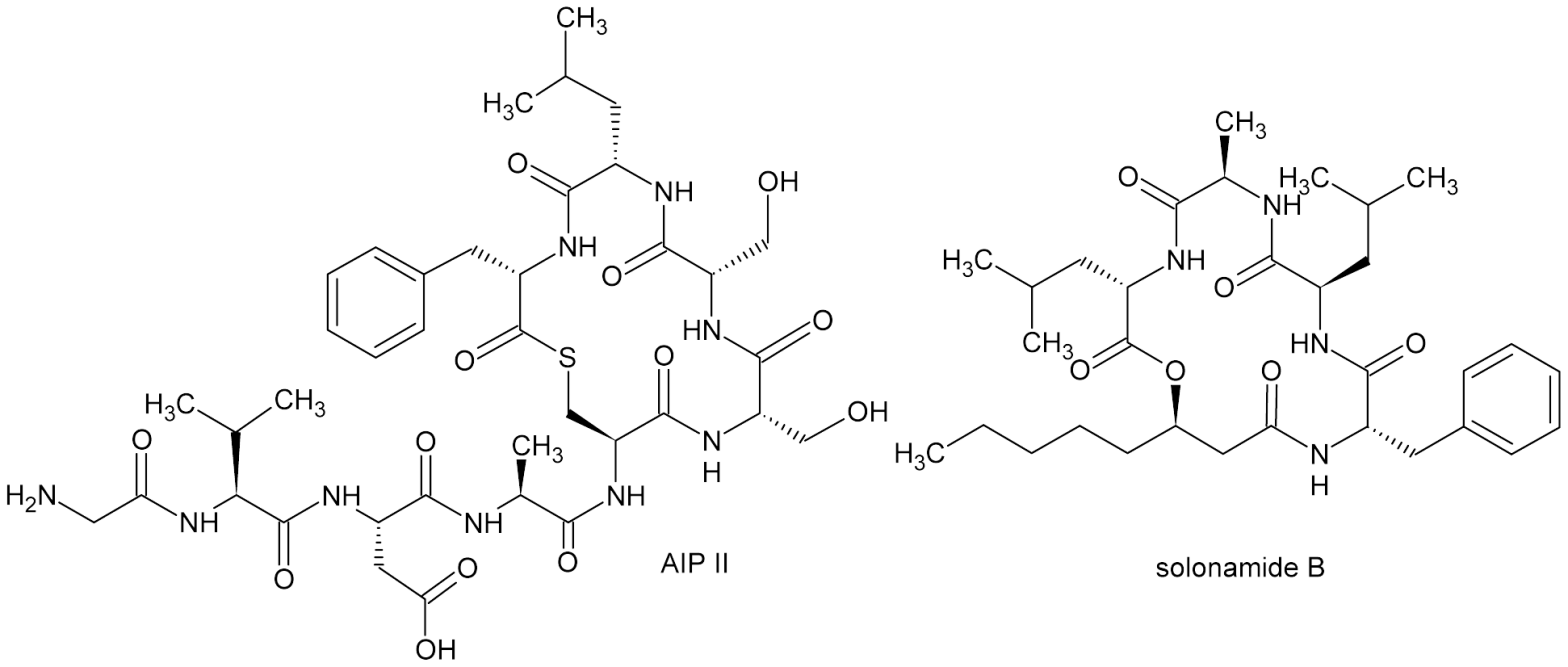
Structures of AIP II and Solonamide B. AIP II (left), Solonamide B (right).

**Figure 5:**
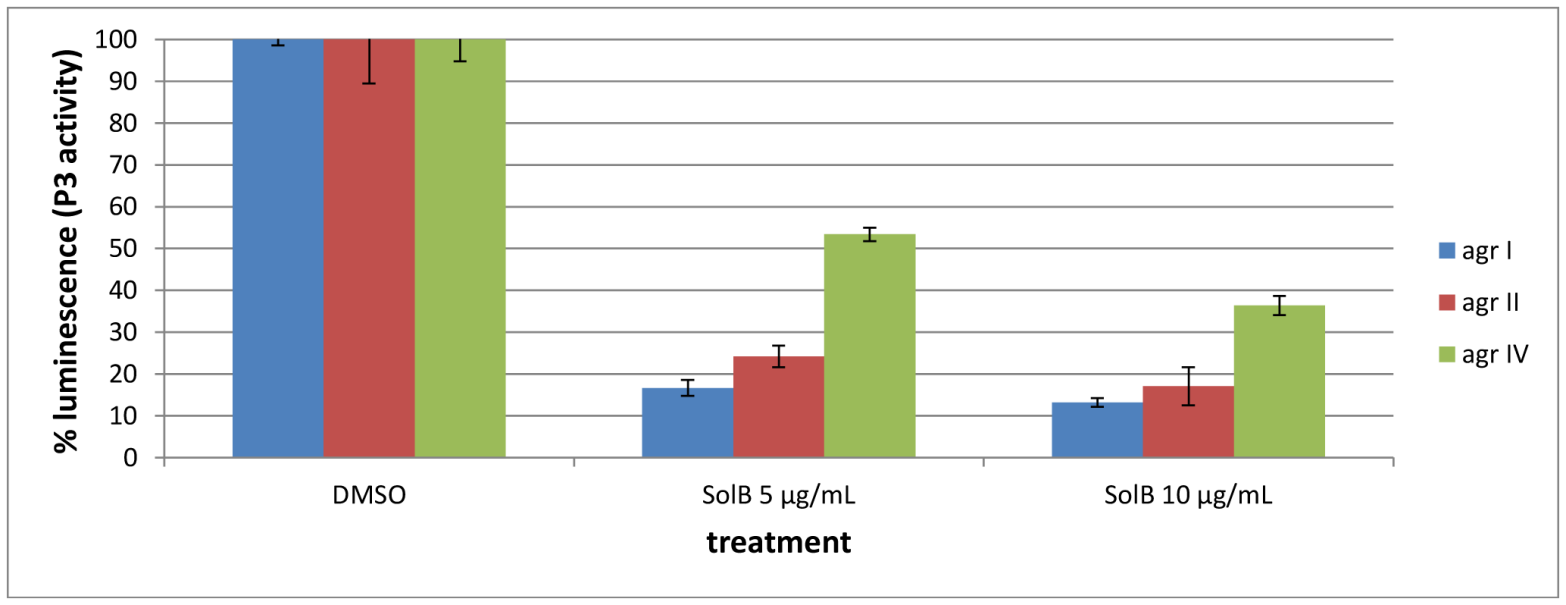
Solonamide B inhibits *agr*-group I, II and IV. The P3::*lux* reporter strains AN1 *agr* I, AN2 *agr* II, AN3 *agrIII* and *agr* IV AN4 were grown with DMSO (control), Solonamide B 5 µg/mL, and Solonamide B 10 µg/mL. Luminescence was recorded after 2½ hours of growth in the presence of the compound. The luminescence is scored in % luminescence relative to the control (DMSO) set to 100% for each *agr* group individually and standard deviations are calculated based on three replicates.

Interestingly, the cyclic structure of Solonamide B resembles the backbone of the native AIPs (fig. 4) that are thiolactones containing seven to nine amino acid residues in which the thiol of the central cysteine is linked to the alpha-carboxyl of the C-terminal amino acid residue [34], [41]. As the thiolactone ring of AIPs is involved in *agr* repression while the peptide tails are linked to activation [42] we examined if Solonamide B inhibits more than one AIP specificity group. To this end we grew *S. aureus P3::lux* reporter strains belonging to the different *agr* specificity groups in the presence or absence of Solonamide B and measured luminescence after 2½ hr incubation at OD_600_ = 0.3 in the microtiter plate. As observed in fig. 5 luminescence expressed by the P3 promoter for *agr* group I and II, was reduced 5 fold when 5 and 10 µg/mL Solonamide B was added compared to the control wells containing DMSO. The strain carrying the *agr* specificity group IV showed a 2–3 fold decrease in *P3::lux* expression when treated with Solonamide B, and the luminescence declined with the higher concentration of Solonamide B. The expression pattern remained unchanged after 3½, 4½, 5½ h and ON incubation (data not shown). For *agr* group III an effect was seen already after 20 minutes resulting in 25% and 35% reduction with 5 and 10 µg/mL Solonamide B, respectively compared to the DMSO control with the effect declining over time (data not shown). Altogether these results show that Solonamide B reduces *agr* expression in all the specificity groups, as measured by P3 activity, with the least effect towards *agr* group III.

### Solonamide B Interferes with Hemolysin Production

**Figure 6:**
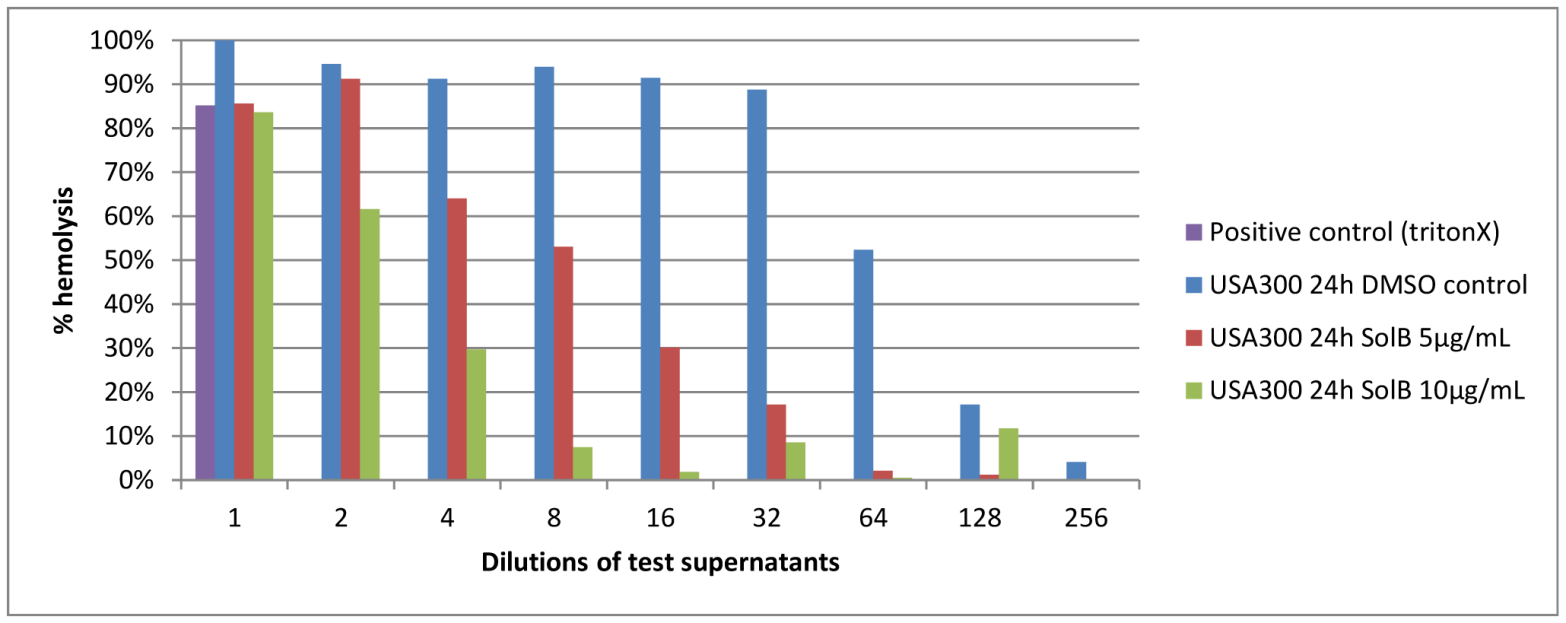
Solonamide B specifically reduces α-hemolysin production. 1–256 fold dilutions of sterile filtered 24 hour culture supernatants from USA300 cultures grown with DMSO, 5 µg/mL or 10 µg/mL of Solonamide B, were tested for its ability to lyse rabbit erythrocytes. 0.1% triton X was used as a positive control. Hemolysis was scored in percentage relative to the DMSO control set to 100%. Data are a representative of at least two independent, biological replicates.

Hemolysis is one of the major virulence factors in *S. aureus* pathogenesis [43]. As we had previously seen that *hla* transcription was strongly reduced by Solonamide B [34] we sought to confirm that hemolytic activity was also affected. We chose to use rabbit erythrocytes for the hemolysis assay as they are known to be particularly susceptible to α-hemolysin [44], [45]. When testing supernatants from *S. aureus* cultures grown with and without Solonamide B on rabbit erythrocytes we observed that Solonamide B reduced production of hemolysin(s) in *S. aureus* strain USA300 (fig. 6). In contrast, the solvent DMSO did not inhibit hemolysin production (fig. 6). When comparing the lytic activities, 50% hemolysis of rabbit erythrocytes was observed with a 64 fold dilution of the USA300 culture supernatants whereas it was seen for an eight fold dilution of supernatant from USA300 cells treated with 5 µg/mL solonamide B and at a three fold dilution for those treated with 10 µg/mL solonamide B. Thus, hemolysin activity is reduced in the presence of Solonamide B.

### Solonamide B Reduces *psmα* Transcription

**Figure 7:**
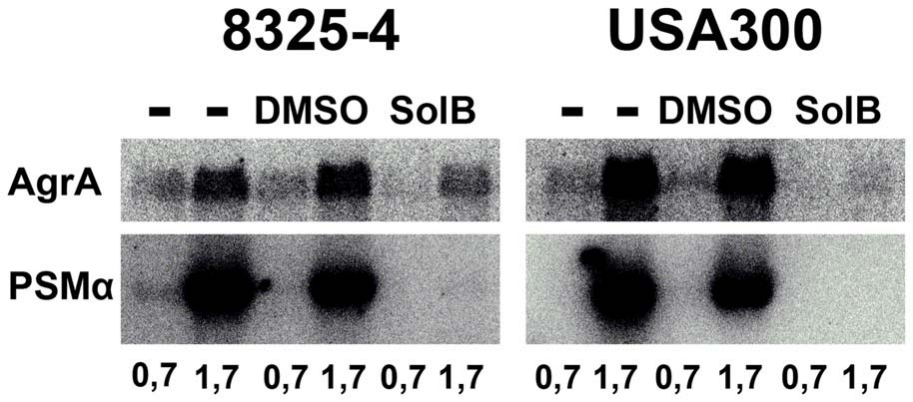
Solonamide B reduces *psmα* and *agrA* expression. Strains USA300 or 8325-4 were grown exponentially to OD_600_ = 0.4 with either 5 µg/mL Solonamide B, DMSO or nothing added. RNA was purified from samples collected at OD_600_ = 0.7 and 1.7, and analyzed by Northern blotting. The membrane was probed with radioactive labeled probes targeting *agrA* and *psmα*.

The PSM phenol soluble modulins are implicated as key virulence factors of CA-MRSA such as USA300 and in contrast to most *S. aureus* virulence genes, their expression is controlled directly by the AgrA response regulator [23]. To determine the potential of Solonamide B as an anti-virulence compound targeting CA-MRSA strains, we examined the effect of Solonamide B on the transcription of the *psm*α operon encoding the alpha-type PSMs. Importantly, when bacterial cells were cultured in the presence of Solonamide B there was a dramatic effect on *psm*α expression as the presence of the compound essentially abolished expression in both strain 8325-4 and USA300 (fig. 7). To assess if this effect is correlated with *agrA* expression we probed the *agr* P2 transcript with a probe covering *agrA* and found it to be significantly decreased in both strains but most so in USA300. Thus, the reduced expression of *psm*α is likely caused by reduced expression of *agrA*.

### Solonamide B Reduces PSM Expression and Neutrophil Killing

**Figure 8:**
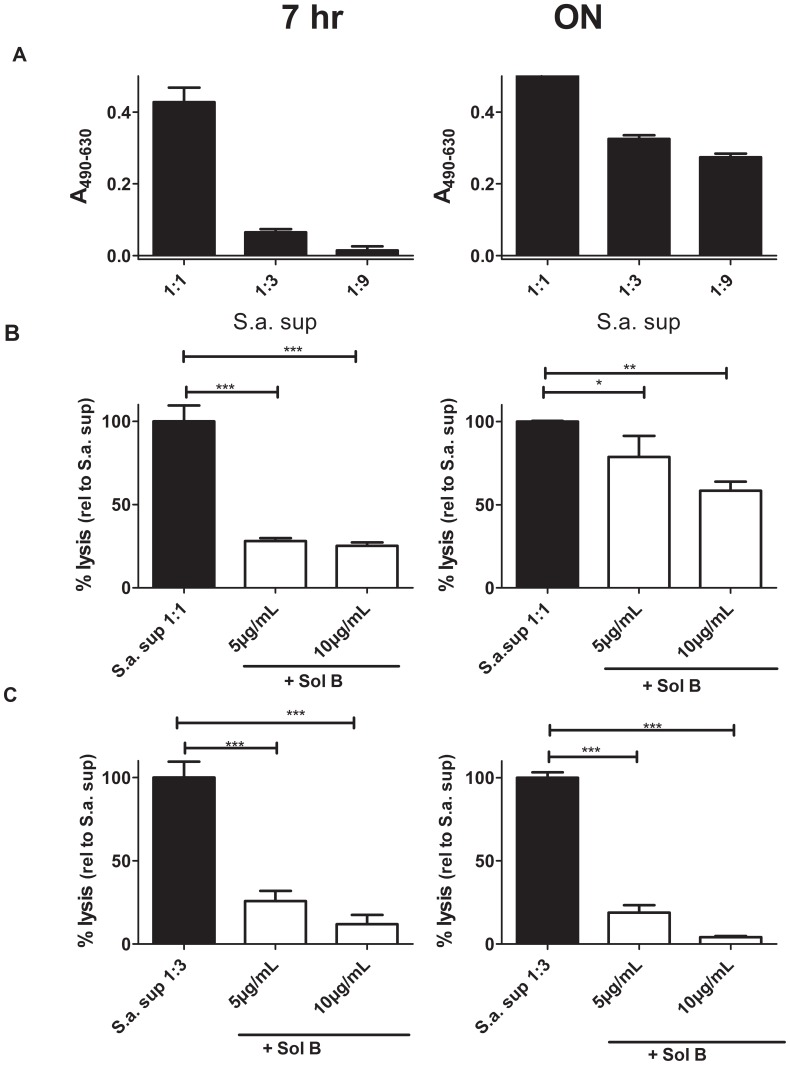
Solonamide B protects against *S. aureus* mediated neutrophil lysis. Sterile filtered supernatants of *S. aureus* USA300 (S.a.) cultures grown for 7 hours or overnight (ON) with either Solonamide B at 5 µg/mL or 10 µg/mL or DMSO (control) were added to isolated human neutrophils. The control supernatant where only DMSO was added was tested in 1-fold, 3-fold and 9-fold dilutions (A). Undiluted (1∶1) supernatants from Solonamide B (SolB) treated cultures are shown together with the control in (B) and 3-fold dilutions (1∶3) are shown in (C). Lysis was monitored by lactate dehydrogenase (LDH) release. Data represents 3 independent experiments, using the average of triplicate wells from each experiment. Asteriks indicate SolB treated cultures resulting in lysis statistically significant from the corresponding control. *, p<0.05, **, p<0.01; ***, p<0.001.

Neutrophils are a part of the innate immune system, and as the first leukocytes that infiltrate affected tissues they are one of the primary defenses against Staphylococcal infection [46], [47]. *S. aureus* produces PSMs of which the alpha type PSM is the most important for neutrophil lysis, with PSMα3 having the most pronounced effect [11], [23]. Since our transcriptional analysis revealed that Solonamide B dramatically reduces expression of *psm*α we examined if it may be protective of *S. aureus* mediated neutrophil killing. To this end we monitored toxicity of sterile filtered CA-MRSA USA300 supernatant grown with and without Solonamide B on human neutrophils (Fig. 8). As above, growth rate and maximum cell density of *S. aureus* was identical with and without Solonamide B (data not shown). Importantly, we found that Solonamide B offered a significant reduction in the toxicity of 7 h and ON culture supernatants of USA300. Thus, the transcriptional effects on both *hla* and *psm*α are translated into reduced toxin production; though other factors than PSM may contribute to neutrophil lysis as well.

During these studies we did not observe lysis of human neutrophils or of bovine erythrocytes at any of the tested concentrations (up to 20 µg/mL) of Solonamide B thus indicating that Solonamide B displays low toxicity (data not shown).

## Discussion

In times where antibiotic resistance is evolving towards most known antibiotics and the development of new antibiotics is lacking far behind we are searching for alternatives to treat serious infectious diseases. One approach that has received considerable interest is anti-virulence therapy where the virulence of the pathogen is targeted rather than viability. In this context we recently isolated a compound from the marine bacterium *Photobacterium halotolerans* that interfered with virulence gene expression in *S. aureus*. The compound termed Solonamide B inversely affected transcription of *hla* and *spa* indicating that it interferes with activation of the *agr* quorum sensing system [34]. We have addressed this hypothesis further by investigating the effect of Solonamide B in mutant cells expressing a variant of the AgrC sensor histidine kinase that is active irrespectively of the presence of the AIP auto-inducing peptides [14]. In this strain background the expression of *rnaIII* was unaffected by the compound strongly indicating that Solonamide B interferes with virulence gene expression by compromising AgrC activation and thus, quorum sensing.

A characteristic property of the *S. aureus agr* system is the presence of at least four *agr* subclasses, in which an AIP from one class induces *agr* in strains of its own class but represses *agr* of the other subclasses [18], [48]. Structural studies of the AIPs have revealed that the macrocyclic ring of the AIPs is responsible for inhibition of *agr*, while the peptide tail is required for *agr*-activation [18], [49]. It has been shown that truncated versions of group II AIPs inhibit all four *agr* groups, and that AIP analogs with oxygen or nitrogen in place of sulfur in the ring structure (i.e., the lactone or lactam vs. the thiolactone) are potent intergroup inhibitors even though they neither activate nor inhibit their cognate receptors [50]. Like the AIPs, Solonamide B is a non-ribosomal peptide with a 16-membered core macrocyclic ring. Both compounds are depsipeptides, however whereas Solonamide B is a lactone, AIP is a thiolactone. Thus, the overall size of the cyclic moiety is quite similar with the peptide tail of the AIPs being replaced by a hydroxy-fatty acid in Solonamide B [34]. When examining Solonamide B we found that it interfered well with *agr* activation in three of the four known classes of *agr* and had a minor effect on *agr* group III. The ability to interfere with *agr* of several subclasses correlates with the cyclic structure of the compound, and the differences observed may be related to the individual AIP structures or the temporal RNAIII induction pattern [51].

*S. aureus* is a versatile pathogen and in recent years the CA-MRSA strains such as USA300 have caused particular concern as they are known for their ability to spread and cause severe infections in otherwise healthy people [9], [36], [52]. Among the virulence factors contributing to the success of these strains is efficient *agr* activation including high expression levels of α-hemolysin as well as the production of the phenol soluble modulins (PSMs) [14]. Neutrophils are one of the primary host defenses against *S. aureus* infections and therefore, neutrophil lysis is important for the virulence of these strains [11]. Importantly we found that Solonamide B dramatically reduces transcription of *psmα* in CA-MRSA strain USA300 as well as the overall toxicity of the culture supernatants when tested against human neutrophils. In contrast to many other virulence factors in *S. aureus* the expression of PSMs is directly controlled by the response regulator, AgrA [23] and also the transcription of *agrA* was significantly reduced by the Solonamide B. Thus our data show that Solonamide B not only interferes with production of virulence factors belonging to the RNAIII regulon but also expression of *agrA* and the AgrA controlled PSMs.

When comparing the ability of Solonamide B to compete with AIP for binding we found that 5 µg/mL solonamide corresponding to 8.5 µM provide 50% inhibition in spent medium of wild type cells containing approximately 0.25 µM AIP (estimated as 5% of 5 µM, Alexander Horswill, personal communication). Thus, although we with the examined compound see a significant reduction in *S. aureus* virulence, structural optimizations may increase the inhibitory potential even further. In conclusion we show that Solonamide B interferes with *agr* activation by binding to the AgrC sensor histidine kinase and thereby preventing interactions between AgrC and the AIPs. As an anti-virulence compound Solonamide B demonstrates potential due to its low toxicity, its inhibitory effect towards the known classes of *agr* and its ability to reduce expression of the PSMs involved in the severe CA-MRSA infections.
